# Acknowledgment to Reviewers of *Marine Drugs* in 2020

**DOI:** 10.3390/md19020045

**Published:** 2021-01-20

**Authors:** Marine Drugs Editorial Office

**Affiliations:** MDPI AG, St. Alban-Anlage 66, 4052 Basel, Switzerland

Peer review is the driving force of journal development, and reviewers are gatekeepers who ensure that *Marine Drugs* maintains its standards for the high quality of its published papers. Thanks to the cooperation of our reviewers, in 2020, the median time to first decision was 13 days and the median time to publication was 32 days. The editors would like to express their sincere gratitude to the following reviewers for their precious time and dedication, regardless of whether the papers were finally published:
Abad-Fuentes, AntonioLópez, Lucía MéndezAbdelmohsen, Usama RamadanLopez, VictorAbedini-Nassab, RoozbehLópez-Cebral, RitaAckerman, MatthewLópez-López, ManuelAcquaviva, RosariaLorberboum-Galski, HayaAdams, DavidLordan, RonanAdams, JamesLorenzo, JuliaAdamski, ZbigniewLorenzo-Morales, JacobAdunyah, Samuel E.Loret, Erwann P.Aflalo, ClaudeLouro, RicardoAfonso, Clélia NevesLowell, AndrewAgòcs, AttilaLu, Jeng-WeiAguilera Correa, John JairoLu, JunAhmad, ShakilLuca, FalzoneAirs, Ruth LouiseLuduena, RichardAkhlaghi, Seyedeh ParinazLuesch, HendrikAleu, JosefinaLund, MarianneAlloatti, GiuseppeLund, Marie BraadAl-Mourabit, AliLutfalla, GeorgesAlonso, EvaLuzina, OlgaAlonso-Padilla, JulioLyukmanova, Ekaterina N.Altamimi, M.Maccallini, CristinaAmaro, HelenaMacias Sanchez, Antonio JoséAmillis, SotirisMaciej, StrzemskiAminin, DmitryMaeda, HayatoAmmazzalorso, AlessandraMaeda, KazuhisaAmorós, AsunciónMagarlamov, Timur Yu.Amoutzias, GrigorisMagnani, MauroAn, Jeung HeeMahar, RohitAnanieva, ElitsaMaher, PamelaAndolfi, AnnaMaione, FrancescoAndrade, Paula B.Maisetta, GiuseppantonioAndreev, Yaroslav A.Majireck, MaxAndrianasolo, EricMajolino, DomenicoAngeli, AndreaMajtan, JurajAngelino, DonatoMajumder, KaustavAngelov, BorislavMakarska-Bialokoz, MagdalenaAntolin, Albert A.Maki, JamesAntonio Malfa, GiuseppeMakvandi, PooyanAntony, Benny S EathakkattuMalfa, Giuseppe AntonioAntunes, AgostinhoMalhão, FernandaArai, MasayoshiMalik, AdeelArana, InesMallis, PanagiotisAranaz Corral, InmaculadaMaloney, KatherineAraniciu, CătălinMalwal, Satish R.Arcidiacono, BiagioMalyarenko (Vishchuk), Olesya S.Arciello, AngelaMalyarenko, Timofey V.Arellano, Juan B.Mancini, InesArteaga, Jesús FernándezMándi, AttilaArtyukhin, AlexManetti, DinaAubourg, SantiagoMangiatordi, Giuseppe FeliceAuh, Joong-HyuckMangoni, AlfonsoAvallone, SylvieMannino, Anna MariaAviles, Francesc XavierManolakis, IoannisAyciriex, SophieMansoorabadi, Steven O.Azam, Aa HaerumanMantzioris, EvangelineAzam, LaylaMarceau, FrançoisAzemar, FabriceMarchev, AndreyAzuma, KazuoMarginǎ, DenisaBaca, Justin T.Márialigeti, KárolyBadino, PaolaMarini, Herbert RyanBagshaw, Stephen A.Marino, AngelaBahn, AndrewMarinov, Georgi K.Bakare, OladapoMarinova, KrastankaBaker, BillMarra, AlbertoBakovic, MaricaMartin, JessicaBalabanova, LarissaMartin, PatrickBaldisserotto, AnnaMartinet, NadineBallante, FlavioMartínez Del Pozo, ÁlvaroBandarra, Narcisa M.Martínez-Abad, AntonioBanerjee, AbhinandanMartínez-Espinosa, Rosa MaríaBanerjee, Hirendra NathMartínez-Poveda, BeatrizBankova, VassyaMartins, AlbinoBanoub, Joe H.Martins, EvaBanskota, Arjun H.Martins, NataliaBarajas, MiguelMartins, RosarioBarakh Ali, Sogra FathimaMartins, SoniaBarba, Francisco J.Martorana, AlessandroBarbaglio, AliceMaruyama, HirokoBarbosa, MarianaMaruyama, KeiBarinova, SophiaMarverti, GaetanoBarker, DavidMasłyk, MaciejBarnathan, GillesMastinu, AndreaBart, Hans-JörgMasullo, MariorosarioBartolotti, MassimoMateos, RaquelBartosz, GrzegorzMathews, IrimpanBartual, AnaMatichenkov, VladimirBasisty, NathanMatos, Ana RitaBaskaran, RathinasamyMatsuda, HisashiBasso, KariMatsugo, SeiichiBatista, PatríciaMaturavongsadit, PanitaBaumann, MarcusMauro, NicolòBeach, DanielMáximo, PatríciaBearne, Stephen L.Mazur-Bialy, AgnieszkaBeccalli, Egle M.Mazur-Marzec, HannaBechthold, AndreasMcCall, Laura-IsobelBeckel, Jonathan M.McCarron, PearseBecker, Daniel P.Mccarthy, PeterBeedessee, GirishMcreynolds, Katherine D.Beemelmanns, ChristineMebs, DietrichBehan, James A.Meder, LydiaBekhit, Alaa El-Din A.Mediero, AránzazuBellmann-Sickert, KathrinMedvedev, Ilya NikolaevichBello, ClaudiaMeghea, AureliaBerdyshev, Evgeny V.Mehariya, SanjeetBerezovsky, IgorMehiri, MohamedBergès, ThierryMehrshad, MalihehBerillis, PanagiotisMelo, TâniaBermudez, MarcelMeneguzzo, FrancescoBertaccini, AssuntaMeng, MenghsiaoBertelloni, FabrizioMenna, MarialuisaBertin, Matthew J.Menzel, LorenzoBertini, LauraMertas, AnnaBezirtzoglou, EugeniaMešić, ArminBhattacharya, SupriyoMetcalf, JamesBiagi, MarcoMeyer, Anne S.Biagini, GiuseppeMi, Fwu-LongBianco, ArmandodorianoMicael, JoanaBianco, GiulianaMichaud, PhilippeBianco, Ismael DaríoMicheau, OlivierBiasucci, GiacomoMickel, StuartBigler, LaurentMikkelsen, MariaBills, Gerald F.Milanova, AneliyaBilski, JanMilewski, SławomirBissoyi, AkalabyaMiller, John H.Bitto, AlessandraMiller, MattBjarne, LandfaldMills, KenBjørn-Yoshimoto, Walden E.Milthorpe, BruceBlaby-Haas, Crysten E.Mimaki, YoshihiroBlanco, AngelesMinami, AkiraBlanco, MariaMinato, Ken-ichiroBloch, SylwiaMiotla, PawelBobasov, EvgeniiMiranda, CristobalBobiński, MarcinMitaine-Offer, Anne-ClaireBocian, SzymonMitchell, AndrewBodi, DorinaMitchell, MiguelBogni, AlessiaMitchell, Scott G.Bohn, TorstenMitrofanova, AllaBoichuk, SergeiMitsuaki, MoriyamaBölcskei, HedvigMiyake, MasaoBontempo, PaolaMiyaoka, HiroakiBorcan, FlorinMizuno, MasashiBorradaile, Nica M.Mizuno, TooruBortner, Carl D.Modica, Maria VittoriaBose, TanimaMogielnicki, AndrzejBoskovic, ZarkoMohankumar, KumaravelBothwell, JohnMoiseyev, GennadiyBouaïcha, NoureddineMokrejš, PavelBoudreau, Paul D.Moldovan, MirelaBougis, Pierre E.Molgó, JordiBourgoin, SylvainMolinaro, AntonioBourguet-Kondracki, Marie LiseMoloney, MarkBovee, Toine F. H.Momekov, GregoriBowden, BruceMomm, FelixBoysen, ReinhardMonchaud, DavidBracco, EnricoMong, Mei-ChinBracher, FranzMontalvo-Rodríguez, RafaelBrash, AlanMontana, Angel M.Brasinika, DespinaMontenegro, LuciaBraun, VolkmarMorales, DiegoBravo, Susana B.Moran, YehuBresson-Bepoldin, LaurenceMorganti, PierfrancescoBroering, RuthMori, TetsushiBrown, DennisMorimura, ShigeruBrown, LindsayMorla, ShravanBrozovic, AnamariaMorzycki, Jacek W.Brunetti, LuigiMouga, TeresaBruzzone, SantinaMouncey, Nigel J.Budny, MarcinMount, AndrewBugni, TimMousley, CarlBühling, FrankMozos, IoanaBuján Gómez, NoemíMrozik, AgnieszkaBurger, CristofMuceniece, RutaBussmann, Rainer W.Mukai, HidehitoButaye, PatrickMulder, Monique TButu, AlinaMüller, Werner E. G.Byun, SanguineMulloy, BarbaraCaba, OctavioMuntean, Dana MariaCabral, JaydeeMurakami, KazumaCacciotti, IlariaMuramoto, KojiCaetano, NídiaMurata, KazuyaCaffrey, PatrickMurata, KenjiCakstina, IneseMurias, MarekCalabria, DonatoMurphy, René J. L.Calado, RicardoMurry, Daryl J.Caldara, MarinaMurugaiyan, JayaseelanCalina, DanielaMuzio, GiulianaCalvano, Cosima DamianaMysliveček, JaromírCampillo, NataliaNagai, HiroshiCampos, FernandoNagai, TakeshiCañada, FranciscoNagamine, TakeakiCancio, IbonNair, Meera SurendranCannalire, RolandoNakagawa, PabloCao, JianNakagomi, TakayukiCapasso, ClementeNakai, RyosukeCapon, RobertNakamura, MotokiCappariello, AlfredoNakamura, ShinsukeCaputa, MichałNakamura, YoshiyukiCarafa, VincenzoNakatani, YoshihikoCaramujo, Maria JoséNakatani, YoshioCarballal, Ramon NovoaNakazawa, MitsuruCarda, MiguelNakazawa, TakehitoCardoso, SusanaNam, Sang-JipCardullo, NunzioNamgaladze, DmitryCarmeli, ShmuelNanjappa, DeepakCARO, YanisNapolitano, AlessandraCarrieri, AntonioNarasimhulu, Chandrakala AlugantiCarrillo, CeliaNaruishi, KojiCaruntu, ConstantinNashmi, RaadCaruso, GabriellaNassar, Zeyad D.Carvalho, PauloNavarra, MicheleCasalino, ElisabettaNazar, Ross N.Casamassimi, AmeliaNedialkov, Paraskev T.Casapullo, AgostinoNedoluzhko, ArtemCasas, FrançoisNees, MatthiasCasero, María CristinaNeri, SimonaCasertano, MarcelloNeugebauer, WitoldCasillo, AngelaNewman, David J.Cassidy, Pamela BondNguyen, BinhCastagna, MichelaNguyen, Hanh ThuyCastellano, ImmacolataNicoletti, RosarioCastro, Juan C.Nicoletti, VincenzoCatani, MartinaNicosia, AldoCatanzaro, ElenaNiedziela, TomaszCatarino, Marcelo D.Nielsen, Peter EigilCattò, CristinaNieto, GemaCatucci, GianlucaNifantiev, NikolayCaulier, GuillaumeNimalaratne, ChamilaCavalu, SimonaNishi, KosukeCecarini, ValentinaNormark, BenjaminCesaro, AnnabelleNotbohm, HolgerChakrabarti, SubhadeepNouioui, ImenChandra-Hioe, Maria VeronicaNovelli, AntonelloChandy, Kanianthara GeorgeNovio, FernandoChang, Hsueh-WeiNowak-Sliwinska, PatrycjaChang, Yung-FuNunzianna, DotiChao, Chih-HuaNuzzo, GenoveffaChao, Louis KuopingNybo, EricCharoensiddhi, SuvimolO’Doherty, GeorgeChaubet, FrédéricO’Harte, FinbarrChauhan, BhareshOakhill, JonChen, Chun-ChiOda, TatsuyaChen, Chung-HwanOgawa, AkikoChen, Chun-HanOgórek, RafałChen, Chun-LinOh, Dong-ChanChen, Jing-HsienOh, Jae-WookChen, RitaOh, Ki-BongChen, TingtingOhshiro, TakashiChen, YiliangOhta, ShinjiChénais, BenoitOhya, SusumuChetwynd, AndyOhya, YoshikazuChhabra, GaganOkamura, YoshikoChianese, GiuseppinaOkazawa, AtsushiChimienti, GiovanniOkino, TatsufumiChiu, Tsai-HsinOlejnik, AnnaCho, Jae YoulOliveira, HélderCho, YongyeonOnduka, ToshimitsuChoi, HojungOng, Eng ShiChoi, HyukjaeOniga, IlioaraChoi, Jong IlOniszczuk, AnnaChoi, Young HaeOrdoñez, LaraChoi, Yung-HyunOrsi, William D.Choshi, TominariOrtega, María JesúsChoudhary, HemantOsborn, HelenChronopoulou, EvangeliaOshiro, NaomasaChrysanthopoulou, AkriviOstrowska, KingaChu, XiangpingOthman, Eman M.Chugunov, Anton O.Otsuka, YuzuruChung, Sang J.Ovchinnikova, Tatiana V.Chwalek, KarolinaOvery, David P.Chworos, ArkadiuszPacifico, SeverinaCiarcia, RobertoPaiva, LiseteCiccone, LidiaPal, SangitaCicero, ArrigoPalanisamy, SatheeshkumarCichero, ElenaPalumbo, AnnaCima, FrancescaPanchal, Sunil K.Ciobica, AlinPanfoli, IsabellaCisowsky, JaroslawPapakyriakou, AthanasiosClanchy, FelixPapapolymeroua, Georgios A. P.Clark, RichardParisini, EmilioClarke, AnthonyPark, ChulhwanCollet, TrudiPark, Hee-SooCollins, Stuart G.Park, Hyun HoColon Saez, JosePark, KyeongsoonColuccia, MauroPark, Kyoung-ChanCongestri, RobertaPark, Soo JeConlon, MichaelPark, WansuConsole, LaraParlesak, AlexandrCopolovici, Dana MariaParnell, Laura K. S.Coppola, DanielaPascal, Steven M.Correa, ArkaitzPasqualetti, MarcellaCorreia-da-Silva, MartaPassarella, DanieleCorsaro, Maria MichelaPassos, MarisaCosta Moutinho, FabíolaPateiro, MirianCosta, ElisabetePatil, AnujaCosta, JoanaPatruno, AntoniaCosta, MargaridaPatterson, JenniferCosta, Pedro M.Pavic, ValentinaCosta, RuiPavlov, AtanasCostantino, ValeriaPavon-Djavid, GracielaCote, PatricePedersen, Kim B.Couderc, FrançoisPegan, Scott D.Couzinet-Mossion, AureliePeigneur, SteveCoyne, KathrynPeirang, CaoCremades Ugarte, JavierPeleteiro, MercedesCristea, CasteliaPelin, MarcoCrossman, LisaPeltomaa, ElinaCrupi, PasqualePembroke, J.TonyCueto, MercedesPeng, Yi-JenCui, YePenissi, Alicia B.Cui, ZhengPepi, MilvaCuif, Jean-PierrePerdih, FrancCurtis, Michael J.Pereira, DavidCutignano, AdelePereira, José AugustoCzernel, GrzegorzPereira, LeonelCzerwinska, MonikaPereira, Olívia R.D’Agostino, PaulPereira, RenatoD’Angelo, MichelePerez, Juan J.D’auria, ValeriaPerez, Lark J.D’Erme, MariaPérez-Parallé Mera, María LuzDahiya, RajivPérez-Pascual, DavidDaly, NorellePerin, GiorgioDamiano, FabrizioPescitelli, GennaroDanikiewicz, WitoldPetcherski, AntonDarafsheh, ArashPetek, SylvainDaranas, Antonio HernándezPeter, MartinDarius, Hélène TaianaPetrásková, LucieDaudé, DavidPetrova, MarinaDavidson, Victor LPhan, Chin-SoonDavis, Keith R.Philipp, ManfredDawood, Mahmoud A. O.Picot, L.De Geest, BartPiggott, AndrewDe Marco, ArioPinto, Diana CláudiaDe Mieri, MariaPiprek, Rafal P.De Oliveira, José Miguel P. FerreiraPisklak, Dariusz MaciejDe Paz, Jose LuisPiunova, VictoriaDe Rosa, Maria CristinaPlastina, PierluigiDe Winter, HansPlavec, JanezDe Zoysa, MahanamaPletzer, DanielDebela, Ahmed M.Plonka, PrzemyslawDekhtyar, JurijsPluskal, TomášDel Pozo, CarlosPogocki, DariuszDelattre, CédricPohl, ElenaDelbarre Ladrat, ChristinePolticelli, FabioDembitsky, Valery MikhailPomin, VitorDesbois, Andrew P.Ponce-Vargas, MiguelDeslandes, EricPopova, Milena P.Desmaële, DidierPorzel, AndreaDevkota, Hari PrasadPotaczek, Daniel PDey, KamolPoterszman, ArnaudDi Buduo, ChristianPoulin, RemingtonDi Dato, ValeriaPourzand, ChararehDi Donato, PaolaPowell, HeatherDi Liegro, ItaliaPozharitskaya, Olga N.Di Natale, ConcettaPozzi, CeciliaDi Paola, LuisaPozzi, SamanthaDi Rosa, MichelinoPozzolini, MarinaDiana, PatriziaPracharova, JitkaDiaz, FernandoPrado-Cabrero, AlfonsoDiaz-Marrero, Ana R.Pratsinis, HarrisDíaz-Ortiz, ÁngelPrentis, PeterDijkstra, Bauke W.Pritzker, KennethDilokpimol, AdipholProchon, MiroslawaDima, Stefan-OvidiuProksch, PeterDing, YousongPrzekora, AgataDinos, GeorgiosPucciarelli, SandraDionysopoulos, DimitriosPuglisi, Melany P.DiTacchio, LucianoPyka, AlinaDocea, Anca OanaQin, SanboDoda, Sai ReddyQuaglino, DanielaDolmatova, Lyudmila S.Radaram, BhaskerDołowy, MałgorzataRadisavljevic, ZivDolzhenko, Anton V.Rafael, DianaDomingues, RosarioRagg, EnzioDomínguez, HerminiaRahman, M. AzizurDomínguez-Álvarez, EnriqueRahme, KamilDomínguez-Pérez, DanyRaina, SatishDonato, RosarioRaja, RathinamDonohue, TerrenceRajauria, GauravDoroftei, BogdanRajput, Mrigendra K. S.Dos Santos, João Miguel MarquesRalston, EmilyDouglas, TimothyRamírez, CarmenDračínský, MartinRamirez-Garcia, AndoniDrakos, EliasRapa, MattiaDruszczynska, MagdalenaRateb, MostafaDuarte, NoeliaRathore, Atul S.Duggan, Brendan M.Ravaioli, StefanoDumbrava, AncaReed, May J.Durazzo, AlessandraReeve, VivienneDutartre, PatrickReich, MarlisDuzgunes, NejatReid, Christopher W.Dwivedi, ChandraharRevuelta, JuliaDymond, MarcusReyes, FernandoDyshlovoy, Sergey A.Reyes-Batlle, MaríaDzunkova, MariaReymond, Jean-LouisEdwin, De PauwReynisson, JóhannesEfird, Jimmy T.Rhim, Jong-WhanEgami, HiromichiRho, Jung-RaeEid, NabilRialland, MickaëlEiroa, José LuisRibeiro, Artur Jorge Araújo MagalhãesEl-Demerdash, AmrRicciardelli, CarmelaEllinger, BernhardRicciardi, Maria RosariaEngelmann, PeterRiccio, GennaroEppink, MichelRiccio, RaffaeleErmakova, Svetlana P.Rice, Christopher A.Escargueil, AlexandreRicelli, AlessandraEscoté, XavierRienzo, MonicaEsposito, GennaroRijo, PatríciaEsposito, GermanaRimpelová, SilvieEsteban, Ma. AngelesRinge, DagmarEsteve-Romero, JosepRischka, KlausEtich, JuliaRitz, UlrikeFabiani, RobertoRoach, ThomasFakira, Amanda K.Robertson, JeremyFalqué López, ElenaRocha, Djenisa H. A.Fan, JianglinRockel, Jason SFanizzi, Francesco PaoloRodrigo, LuisFaraloni, CeciliaRodrigues, MárcioFarkaš, PavolRodríguez, JaimeFatouros, Dimitrios G.Rodziewicz-Motowidło, SylwiaFaugeron-Girard, CélineRogato, AlessandraFeás, XesúsRoger, JérômeFenical, WilliamRomano, GiovannaFernandes, Carla Sofia GarciaRonsard, LaranceFernández Castro, José JavierRoque, AliciaFernandez-Botran, RafaelRosic, NedeljkaFernando, BinoshaRossi, MiriamFernando, I.P.ShanuraRossiter, SharonFerraboschi, PatriziaRouge, PierreFerraro, VincenzaRoullier, CatherineFerreira, JorgeRoumenina, LubkaFerri, FlaminiaRovini, AmandineFerro, DomenicoRoy, AnuradhaFiedler, Hans-PeterRozalski, MarcinFierascu, Radu ClaudiuRtimi, SamiFigadere, BrunoRubino, Federico MariaFigueira, FlavioRubio Alonso, JuanFigueiredo, JanineRudolf, EmilFiguerola, BlancaRuiz-Velasco, VictorFilice, SimonaRusso, PasqualeFiorillo, LucaRusso, PatriziaFisher, Jed F.Ryan, MarionFitton, HelenRye, Philip D.Flórez-Fernández, NoeliaRyo, UshiodaFocaroli, StefanoRyu, BoMiFogacci, FedericaSabila, PaulFontana, AngeloSacco, PasqualeFontana, FabrizioSadkowski, TomaszFonte, PedroSafavi, HelenaFormato, MarilenaSaha, Manik LalForte, Iris MariaSaha, Sushanta KumarFousteris, ManolisSahu, Saura C.Franco, ChrisSak, Jarosław J.Frank, Craig L.Sakai, RyuichiFrattaruolo, BrLucaSala, Gerardo DellaFroeyen, MatheusSalamanca, Constain H.Fuchs, SabineSalerno, SimonaFujii, RitsukoSalim, AngelaFujimori, KoSalto-Gonzalez, RafaelFujita, MasakiSalvatori, RobertaFujita, MorifumiSamba-Louaka, AscelFujita, YuichiSamhan-Arias, AlejandroFukada, HaruhisaSanchez, LauraFumagalli, LauraSanchez-Amat, AntonioFunamizu, NaotakeSancisi, ValentinaFuruta, KazuyukiSanina, Nina M.Fuwa, HaruikoSanjeet, MehariyaGagaoua, MohammedSanjeewa, Kalu Kapuge AsankaGagat, PrzemyslawSankaranarayanan, Nehru VijiGăină, Luiza IoanaSansone, ClementinaGalindo-Villegas, JorgeSantero, EduardoGálvez, JulioSantos Vizcaíno, EdortaGalvez-Llompart, MariaSantos, RenatoGaly-Fauroux, IsabelleSaponaro, ConcettaGanesan, A.Sarabia, FranciscoGarbayo, InésSaravanakumar, KandasamyGarbuzenko, OlgaŠarkanj, BojanGarcia, AntonioSarkar, DhrubaGarcía-Davis, SaraSastre, LeandroGarcia-Sosa, AlfonsoSastry, JagannadhaGarcia-Vaquero, MarcoSato, EiichiGaro, ElianeSatomi, YoshikoGaul, DavidSaurav, KumarGavagnin, MargheritaSavage, Paul B.Gavaia, PauloSaviano, MicheleGavenonis, JasonSawhney, NehaGazizov, AlmirScandurra, RobertoGe, XiaoweiSchaal, WesleyGebauer, JanSchaeberle, TillGeerlof, ArieScheffel, JörgGeffroy-Rodier, ClaudeSchmalz, Hans-GuentherGenilloud, OlgaSchmidt, EdGennaro, RenatoSchöllhorn, BerndGenovese, GiuseppaSchröder, Heinz C.Genta, IdaSchroeder, ChristinaGentilucci, LucaSchweigkofler, WolfgangGerbaux, PascalSciubba, FabioGeremia, SilvanoScotto D’Abusco, AnnaGesell, AndreasScrima, MarioGhidini, MicheleSebestik, JaroslavGhinet, AlinaSeca, Ana M. L.Ghiulai, RoxanaSecott, TimGhosh, RobinSedic, MirelaGiangrande, AdrianaSeijas Vázquez, Julio A.Gierszewska, MagdalenaSeipke, RyanGil, RosarioSellars, JonathanGillet, GermainSemenycheva, Ludmila L.Giordano, DanielaSenbonmatsu, TakaakiGirelli, Chiara RobertaSengupta, BidishaGiromini, CarlottaSeo, Jung-KilGismondi, AngeloSeo, ShigemiGlaser, KeithSepcic, KristinaGlukhov, EvgeniaSerrano-Aroca, ÁngelGogulamudi, Venkateswara ReddySerreli, GabrieleGoh, Gerard Kian-MengSethi, GautamGomes, NelsonShaaban, Khaled AGomez-Estaca, JoaquinShamil, AfiyatullovGómez-Guzmán, ManuelShang, QingsenGonçalves, Ana M. M.Shang, ZhuoGoncharov, Nikolay V.Sharkawi, TahmerGonec, TomasShavandi, AminGonkowski, SławomirShaw, SubrataGonzalez, Juan M.Shchennikova, AnnaGonzález-García, JorgeShcherbik, Natalia V.Gorbi, StefaniaSheehy, EamonGornowicz, AgnieszkaSheu, Jyh-HorngGrabinska, KarionaShigemura, YasutakaGrad, SibylleShigeyuki, KawaiGrande, RossellaShikov, AlexanderGrant, GeorgeShimada, YasuhitoGray, ChristophertShin, Hee JaeGrechkin, Alexander N.Shin, Hyun-JaeGreen, Paul W. C.Shin, JongheonGrkovic, TanjaShinada, TetsuroGrogan, GideonShirahata, TatsuyaGrose, JulianneShridhar, SurekhaGrossi, GiancarloShugo, SuzukiGrovel, OlivierSicinski, RafalGupta, KajalSidorenko, AlexanderGupta, KuldeepSiebert, Hans-ChristianGuriec, NathalieSikiric, PredragGurikov, PavelSil, SusmitaGushina, IrinaSilipo, AlbaGustafson, KirkSilva, CatarinaGutiérrez-Gallego, RicardoSilva, Elisabete RibeiroGuzow-Krzemińska, BeataSilva, JoanaHabala, LadislavSilva, JoãoHäder, Donat P.Silva, Marcelo SousaHahn, DongyupSilva, MarisaHakkinen, LariSilva, Simone S.Han, Dong-WookSilva, SofiaHan, Se JongSilva, TiagoHannan, Md. AbdulSimões, Marta FilipaHano, ChristopheSimon, JorgeHansen, EspenSimón-Vázquez, RosanaHardy, RowanSimpson, Bradley S.Harris, Jonathan M.Sims, Ian M.Harvey, JoanneSing, Swee LeongHarvey, PetaSingh, AnandHarwood, TimSingh, JonathanHashimoto, MakotoSingh, Puspendra P.Haslinger, KristinaSinnott, Michael L.Hatano, YuichiroSintsova, OksanaHaudecoeur, RomainSiodłak, DawidHaug, TorSipkema, DetmerHavelek, RadimSistla, SrinivasHayashi, YasuakiSivanesan, IyyakkannuHayes, MariaSkellam, ElizabethHeal, KatherineSkorik, Yury A.Heath, TimothySlama, PetrHeneberg, PetrŚliwińska-Wilczewska, SylwiaHenke, AndreasSmiesko, MartinHeo, Soo-JinSmith, DavidHernandez-Prieto, Miguel A.Smith, G. JasonHinds, Terry D.Smith, Thomas E.Hirasaka, KatsuyaSnarr, Brendan D.Hiroaki, HidekazuSnowden, TimothyHo, Yi-ChengSoares, Antonio GarciaHolguin, Francisco OmarSobierajska, KatarzynaHolsæter, Ann MariSobotta, ŁukaszHolz, Richard C.Soderhall, IreneHomma, TakujiroSöderhäll, KennethHong, KuiSoengas, Raquel G.Horgen, F. DavidSolano, FranciscoHosokawa, MasashiSolcan, CarmenHrabal, RichardSolomonov, InnaHsieh, YvesSolovchenko, AlexeiHsing, Chung-HsiSolyanikova, InnaHsu, Fong-FuSone, HidekoHuang, Chun-YungSorg, OlivierHuang, Huey-ChunSosic, AliceHuang, Tse-HungSosio, MargheritaHuber, Eva M.Soundharrajan, IlavenilHumbert, MagaliSovadinova, IvaHung, Hsin-YiSrinivas, JanaswamyHussain, HidayatSserwadda, MartinHwang, Bang YeonStaal, JensIadonisi, AlfonsoStacewicz-Sapuntzakis, MariaIanora, AdriannaSteen, Ida HeleneIatridi, ZacharoulaSteiger, StefanieIbrahim, SalamStenstrøm, YngveIguchi, TaisenStepanic, VisnjaIkonomopoulou, MariaStephen, Michael RajeshIlagan, Ma. Xenia G.Stevens, David ColeIlan, MichaStoskopf, MichaelImam, Mohammad ZafarStrangman, WendyImbimbo, BrunoSu, Wen-TaImperatore, ConcettaSugni, MichelaImperatore, RobertaSugumaran, ManickamInman, Denise M.Suñe Negre, Jose MariaInoue, AkiraSung, Ping-JyunIsaza Martínez, José HipólitoSungthong, RungrochIto, TakuyaSurguchov, AndreiItoh, TakafumiSurup, FrankItta, ArunSutherland, Hamish S.Ivanets, ElenaSuzuki, KatsuhikoIvanova, Donika G.Suzuki, KeikoIvanovska, NinaSuzuki, ToshiyukiIwai, AtsushiSvetashev, VasilyIzzo, IreneSynytsya, AndriyJablonska, EwaSzabo, AndrasJadeja, RavirajsinhSzakiel, AnnaJaganjac, MoranaSzakonyi, ZsoltJakobusic Brala, CvijetaSzczes, AlexandraJallet, DenisSzebeni, Gábor JánosJanczarek, MonikaSzekalska, MartaJanda, KatarzynaSzewczyk, KatarzynaJasutiene, InaSzweda, PiotrJaumot, JoaquimSzymańska, EmiliaJaźwiński, JarosławTae, Han-ShenJe, Jae-YoungTähtinen, PetriJenner, RonaldTaïwe, Germain SotoingJennings, Jessica AmberTakata, JiroJenssen, HåvardTakemura, MihoJeon, TaeckTakeshita, SatoshiJeon, You-JinTakuya, KosekiJeong, DaewonTalero, ElenaJeong, Se KyooTamura, Jun-ichiJimbo, MitsuruTanaka, JunichiJiménez, CarlosTanaka, MasashiJin, JeanTanaka, NaonobuJo, HyunilTanase, CorneliuJokić, StelaTang, Bor LuenJones, ChrisTang, Cheng-MingJones, Jessica L.Tanzi, Maria CristinaJones, Meriel G.Tarantino, GiovanniJoo, Hong-GuTarasova, Ol’Ga A.Jordheim, MonicaTarman, KustiariyahJose, JineyTarrago, LionelJung, SaschaTartaglione, LucianaJurj, Ancuţa MariaTasdemir, DenizKačániová, MiroslavaTashiro, MitsuruKadokawa, Jun-ichiTavakoli, JavadKadota, IsaoTedesco, IdoloKafarski, PawelTelukutla, Srinivasa ReddyKajarabille, NaroaTempesta, MariaKalendová, AlenaTeta, RobertaKalinin, Vladimir I.Teusch, NicoleKalinski, Jarmo-Charles J.Tharmalingam, NagendranKallscheuer, NicolaiTheoduloz, CristinaKamath, SandipThies, StephanKamisuki, ShinjiThomas, OlivierKamiya, MitsunobuThorpe, ClareKanakkanthara, ArunThoury, MathieuKaneko, GenTidgewell, KevinKanrar, SiddharthaTietze, AlesiaKapral, MałgorzataTilocca, BrunoKaran, DevTimoshenko, Alexander V.Karnati, Konda ReddyTinta, TinkaraKarpiński, Tomasz M.Tischler, DirkKarwowski, BoleslawTkaczewska, JoannaKashchenko, Nina I.Tobacman, JoanneKashman, YoelTobiasch, EddaKasprzak, AldonaToepel, JörgKaszowska, MartaTognolini, MassimilianoKatikou, PanagiotaToiu, AncaKatsuyama, YoheiTomaszewska, EwaKavita, KumariTomić, SimonidaKawabata, KyuichiTomoda, HiroshiKawai, TatsuoTondi, DonatellaKawakami, YukiTonello, FiorellaKawamura, AkiraTori, MotooKawamura, FumioTorres Perez, María DoloresKawiak, AnnaTosti, ElisabettaKayumov, AiratTotta, PierangelaKazuhiro, ShikinakaToyoda, MasashiKeereetaweep, JantanaTrammell, SamuelKeith, GordonTran, TrongKeller, AimeeTrandafirescu, Cristina-MariaKellogg, ChristinaTrim, CarolKelly, MichelleTrincone, AntonioKerch, GarryTrofimov, Aleksei V.Kerr, RussellTrojanowicz, BoguszKeshari, SunitaTrujillo-Rodríguez, María JoséKesharwani, SiddharthTsai, Huai-JenKeyzers, RobertTsai, Jen-NingKhachik, FredTsai, Rong-KungKhalil, ZeinabTsatsakis, AristidisKhan, Mohd MohsinTsentalovich, Yuri P.Khozin-Goldberg, InnaTsetlin, Victor I.Khurshid, ChrowTsiafoulis, Constantinos G.Kicel, AgnieszkaTsoupras, AlexandrosKim, BokyungTsumuraya, TakeshiKim, Hak JunTsurkan, Mikhail V.Kim, HangunTudela, JoseKim, HeiTurk, TomKim, Hyeung-RakTurkmen, SerhatKim, Hyun-WooTykarska, EwaKim, InKyeomTytgat, JanKim, JaehanTzeng, Tzuen-RongKim, Jeong-HwanTziveleka, Leto-AikateriniKim, Ji YeonTzovenis, IoannisKim, Jin-KyungUeno, AkioKimura, Ken-ichiUeno, MikinoriKirby, GilesUhrin, DusanKirienko, NatashaUjević, IvanaKiselevskiy, MikhailUlanova, DanaKiss, TivadarUlber, RolandKittakoop, PrasatUmerska, AnitaKiuru, PaulaUngureanu, CameliaKlettner, AlexaUrbain, AurélieKlewicka, ElzbietaUrbatzka, RalphKlijnstra, Mirjam D.Urbinati, GiorgiaKlocko, Amy L.Urzi, ClaraKluczyk, AlicjaUsami, YoshihideKnight, DavidUsov, Anatolii IKo, Kam MingUstyuzhanina, NadezhdaKo, Sung-kyunV Ivanov, AlexanderKogure, NoriyukiVago, RiccardoKojima, ChojiroValado, AnaKokkinidis, MichaelVale, PauloKokkola, TarjaValentao, PatriciaKokou, FotiniValentová, KateřinaKokoulin, Maxim S.Valeriote, FrederickKoksharova, Olga A.Vallesi, AdrianaKoledova, ZuzanaVallini, GiovanniKolesnikova, Sophia A.Vamanu, EmanuelKomaki, HisayukiVan Den Bogaart, GeertKonno, KatsuhiroVan Etten, JamesKontos, Christos K.Vann, WillieKooistra, AlbertVarela, JoãoKootala, SujitVarga, GáborKornienko, AlexanderVasconcelos, Vitor Manuel O.Korolkova, YuliyaVasicek, OndrejKoshino, HiroyukiVavitsas, KonstantinosKotoku, NaoyukiVázquez, José AntonioKourmentza, ConstantinaVega, Jose MariaKoval, OlgaVega-Rubín-De-Celis, SilviaKowalski, RadosławVerestiuc, LilianaKoyama, NobuhiroVerma, RanjitKozlov, SergeyVetvicka, VaclavKozlowska, JustynaViapiana, AgnieszkaKraševec, NadaVicente, FranciscaKreis, WolfgangVil’, Vera A.Křen, VladimirVillalba-Galea, Carlos A.Krock, BerndVincent, BrunoKrüger, MarcusVinciguerra, ManlioKryshchyshyn, Anna P.Vinogradov, EvgueniiKubala, LukášVismara, ElenaKubica, PawełVisnapuu, TriinuKubo, MiwaVodnar, Dan CristianKudo, YutaVolpato, MileneKulkarni, Chandrashekhar V.Von Neuhoff, NilsKulkarni, DhananjayVon Salm, JacquelineKulminskaya, Anna A.Vorob’ev, Mikhail M.Kumagai, YuyaVoronina, O. L.Kumar Jha, MithileshVoshavar, ChandrashekharKumar, AnujVydra, NataliaKumirska, JolantaWada, JunKuo, Ping-ChungWahli, WalterKurakula, MalleshWallace, David R.Kurihara, HideyukiWang, GuankuiKurihara, TatsuoWang, JunjieKurimoto, ShinichiroWang, QunKuroboshi, ManabuWang, YanwenKuryłowicz, AlinaWarren, J. DavidKusaykin, Mikhail I.Watanabe, KenshiKushkevych, IvanWatanabe, RyuichiKwiecien, JacekWeiss, SiegfriedKwok, Henry Hang FaiWelling, MickKwon, Hyuk NamWenta, TomaszKwon, Young M.Whalen, KristenLa Clair, JamesWheeler, ThomasLa Penna, GiovanniWieczorek, Rafal M.La Regina, GiuseppeWiedman, GregoryLaabir, MohamedWietrzyk, JoannaLabro, AlainWilliams, PhilipLacina, LukasWilson, DavidLade, HarshadWilson, RichardLafont, ElodieWindshügel, BjörnLage, SandraWińska, KatarzynaLai, Pin-KuangWise, LynLamas, AlexandreWittemann, AlexanderLancelin, Jean-marcWittine, KarloLang, SiegmundWnuk, MaciejLanger, ThierryWojtunik-Kulesza, Karolina AnnaLappan, UweWong, AlanLaraia, LucaWright, AmyLaroche, CélineWu, BinLatowski, DariuszWu, Chang-JerLauridsen, CharlotteYadav, VinitaLaurienzo, PaolaYamamoto, YusukeLavison-Bompard, GwenaëlleYamazaki, TomomiLazado, Carlo C.Yan, ShengminLe Visage, CatherineYang, Feng-LingLeal, FernandoYang, InhoLee, Chia-HwaYang, Seung HwanLee, Hyi-SeungYang, Shun-ChinLee, Jun SikYano, AkiraLee, Mei-HsienYaoita, YasunoriLee, MinaYasuda, KaoriLee, Myung KooYazdi, ImanLee, Sang KookYen, Frances T.Lee, Sang-HanYeo, SynLee, SanghyunYermak, Irina M.Lee, VictorYing, MingyaoLee, Yeon-JuYokoyama, SaichiroLefranc, FlorenceYoo, Hah YoungLehocký, MariánYoshida, TakashiLeitão, Jorge H.Yoshino, HironoriLengyel, ImreYotsu-Yamashita, MariLeone, AntonellaYoussef, DiaaLeroy, BaptisteYu, Chang YeonLeshchenko, Elena V.Yu, Jae-sikŁęska, BogusławaYuann, Jeu-Ming P.Lesyk, Roman B.Zadvornyy, Oleg A.Leung, IvanhoeZafar, MuhammadLeutou, Alain SimpliceZahradka, PeterLevy-Ontman, OshratZaitsev, AlekseyLewandowska, KatarzynaZakharenko, Alexander M.Lewinska, AnnaZamostny, PetrLewis, RichardZamyatnin, AndreyLeychenko, ElenaZárate, RafaelLi, Hsing-HuiŻarowska, BarbaraLi, WenyiZaucke, FrankLiang, ZhibinZeeshan, MuhammadLiao, Jiunn-WangZehbe, IngeborgLiberek, BeataZelepuga, ElenaLima, SofiaZeman, DanielLin, HaishuZeuner, BirgitteLin, LigenZgórka, GrażynaLin, RuweiZhang, ChangyiLin, Tung-YiZhang, CongqiangLin, YanghsiangZhang, JunzengLindequist, UlrikeZhang, YonghongLindstrom, Jon MartinZhang, YumiaoLittlefield, Bruce A.Zheng, ShilongLiu, ShuangZhou, ShengbinLiu, YangZhu, XuejunLiverani, LilianaZhuravleva, Olesya I.Lizard, GérardZienkiewicz, KrzysztofLlewellyn, Lyndon E.Zilberberg, NoamLo Giudice, AngelinaZitko, JanLogan, SreemathiZnachor, PetrLoiseau, NicolasZoccatelli, GianniLoll, BernhardZubía, EvaLomova, MariaZubkova, OlgaLondoño López, Martha ElenaZuccari, GuendalinaLonghi, GiovannaZuk, MagdalenaLongo, CaterinaZuorro, AntonioLoots, GabrielaZwierzchowski, GrzegorzLopes, Graciliana

